# Evaluation of Tumor Response after Short-Course Radiotherapy and Delayed Surgery for Rectal Cancer

**DOI:** 10.1371/journal.pone.0160732

**Published:** 2016-08-22

**Authors:** Daniela Rega, Biagio Pecori, Dario Scala, Antonio Avallone, Ugo Pace, Antonella Petrillo, Luigi Aloj, Fabiana Tatangelo, Paolo Delrio

**Affiliations:** 1 Colorectal Surgical Oncology, Istituto Nazionale per lo Studio e la Cura dei Tumori–“Fondazione Giovanni Pascale” IRCCS, Naples, Italy; 2 Division of Radiotherapy, Department of Diagnostic Imaging, Radiant and Metabolic Therapy, Istituto Nazionale per lo Studio e la Cura dei Tumori–“Fondazione Giovanni Pascale” IRCCS, Naples, Italy; 3 Division of Gastrointestinal Medical Oncology, Istituto Nazionale per lo Studio e la Cura dei Tumori–“Fondazione Giovanni Pascale” IRCCS, Naples, Italy; 4 Division of Radiology, Department of Diagnostic Imaging, Radiant and Metabolic Therapy, Istituto Nazionale per lo Studio e la Cura dei Tumori–“Fondazione Giovanni Pascale” IRCCS, Naples, Italy; 5 Nuclear Medicine Unit, Istituto Nazionale per lo Studio e la Cura dei Tumori–“Fondazione Giovanni Pascale” IRCCS, Naples, Italy; 6 Pathology Unit, Istituto Nazionale per lo Studio e la Cura dei Tumori–“Fondazione Giovanni Pascale” IRCCS, Naples, Italy; Instituto Nacional de Cancer, BRAZIL

## Abstract

**Purpose:**

Neoadjuvant therapy is able to reduce local recurrence in rectal cancer. Immediate surgery after short course radiotherapy allows only for minimal downstaging. We investigated the effect of delayed surgery after short-course radiotherapy at different time intervals before surgery, in patients affected by rectal cancer.

**Methods:**

From January 2003 to December 2013 sixty-seven patients with the following characteristics have been selected: clinical (c) stage T3N0 ≤ 12 cm from the anal verge and with circumferential resection margin > 5 mm (by magnetic resonance imaging); cT2, any N, < 5 cm from anal verge; and patients facing tumors with enlarged nodes and/or CRM+ve who resulted unfit for chemo-radiation, were also included. Patients underwent preoperative short-course radiotherapy with different interval to surgery were divided in three groups: A (within 6 weeks), B (between 6 and 8 weeks) and C (after more than 8 weeks). Hystopatolgical response to radiotherapy was measured by Mandard’s modified tumor regression grade (TRG).

**Results:**

All patients completed the scheduled treatment. Sixty-six patients underwent surgery. Fifty-three of which (80.3%) received a sphincter saving procedure. Downstaging occurred in 41 cases (62.1%). The analysis of subgroups showed an increasing prevalence of TRG 1–2 prolonging the interval to surgery (group A—16.7%, group B—36.8% and 54.3% in group C; p value 0.023).

**Conclusions:**

Preoperative short-course radiotherapy is able to downstage rectal cancer if surgery is delayed. A higher rate of TRG 1–2 can be obtained if interval to surgery is prolonged to more than 8 weeks.

## Introduction

Preoperative long course radiotherapy has been shown to be effective in downsizing locally advanced rectal tumors [1–3]. Long-course chemo-radiation has been extensively applied and encouraging results derive from this approach in terms of local control with a high rate of tumor regression up to a significant rate of complete response. Short-course radiotherapy (SCR) has been used with a different goal, i.e. “sterilizing” the irradiated area immediately before surgery without any expected on the tumor’s stage and size. This is mainly due to the short overall treatment time (OTT) in patients operated on in the week following the end of the radiotherapy. It is known that delaying surgery after chemo-radiation produces a significant rate of tumor regression [4–8]. Therefore the increase of the OTT (by prolonging the interval between radiation and surgery) could raise the rate of tumor regression and induce a higher rate of complete response even in patients treated with SCR [9–11]. There aren’t many data on the effect on tumor regression due to a prolonged interval after SCR in locally advanced rectal cancer: Pach et al and Stockholm III trial don’t produce significant different with delayed surgery [12]. Tumor regression grade (TRG) is a powerful indicator of response to a neoadjuvant treatment in rectal cancer and can measure the effectiveness of radiation therapy. The aim of this study was to evaluate the TRG as indicator of response to SCR followed by a progressive prolonged interval prior to surgery in patients with middle and distal rectal adenocarcinoma.

## Material and Methods

A dedicated database with all information on 649 rectal cancer patients assessed and treated at our Istitution was retrospectively analysed. Since January 2003 to December 2013 sixty–seven consecutive patients with rectal cancer cT3N0 ≤ 12 cm from the anal verge and with circumferential resection margin (CRM) > 5 mm, evaluated by magnetic resonance imaging (MRI), 1) cT2 any N < 5 cm from anal verge, and 2) tumors with N+ that are unfit for long course chemoradiation, have been treated with preoperative SCR. All patients were discussed into a multidisciplinary case conference: staging and medical comorbidities were considered. In our series we used preoperative short-course radiation as an alternative to CRT in elderly patients or for patients unfit for preoperative chemotherapy due to severe comorbidities (ant T, any N, any CRM). The Karnofsky index was also used to score the patient general conditions and only patients with KPS ≥ 70 were considered eligible for long chemoradiation and here defined as fit for combined neoadjuvant therapy. Surgery alone is performed in early rectal cancer cT1 cN0 cCRM < 5 mm.

During the study period, the interval between the completion of radiation treatment and surgery was progressively prolonged from one to 10 weeks. The increase was defined into three different calendar periods, as depicted in Table 1. Beneficial to a simpler analysis, patients were then divided into three groups: group A (surgery within 6 weeks from SCR), group B (surgery between 6 and 8 weeks from SCR) and group C (surgery after more than 8 weeks from SCR). Patients with rectal cancer underwent a pre-treatment work-up including whole body computer tomography (CT) scan, pelvic MRI and endorectal ultrasound. A positron emission tomography (PET) scan was also performed in most patients before radiation therapy and before surgery to monitor response to treatment. All pre-treatment exams were repeated with the restaging before the planned operation. Downstaging was considered as the reduction of the pathological stage both for tumor and lymphnodes after neoadjuvant therapy.

**Table 1:** Baseline characteristics of the patients grouped according to interval to surgery.

	< 6 wk	6–8 wk	> 8 wk	*p*
	n = 12 (%)	n = 19 (%)	n = 35 (%)	
**Gender**				0.32*
Male	5 (41.7)	13 (68.4)	23 (65.7)	
Female	7 (58.3)	6 (31.6)	12 (34.3)	
**Age (years)**				0.93**
Mean (SD)	69 (12.8)	71 (10.1)	71 (10.7)	
Range	31–80	43–85	49–91	
**Study period**				< 0.001*
2003–2004	9 (75.0)	1 (5.3)	0 (0)	
2005–2008	2 (16.7)	12 (63.2)	4 (11.4)	
2009–2013	1 (8.3)	6 (31.6)	31 (88.6)	
**Stage**				0.10*
I—T_2_N_0_	1 (8.3)	0 (0)	2 (5.7)	
II—T_3_N_0_	11 (91.7)	14 (73.7)	20 (57.1)	
III—T_2-3_N_1-2_	0 (0)	5 (26.3)	13 (37.2)	
**Distance from a.v.**				0.28*
≤ 5 cm	7 (58.3)	9 (47.4)	13 (37.2)	
5–8 cm	5 (41.7)	5 (26.3)	11 (31.4)	
≥ 8 cm	0 (0)	5 (26.3)	11 (31.4)	
**Fitness for long course chemoradiation**				0.008*
Unfit	4 (33.3)	14 (73.7)	11 (31.4)	
Fit	8 (66.7)	5 (26.3)	24 (68.6)	

*exact chi-square test

** Kruskal-Wallis non parametric Analysis of variance

### Radiotherapy

All patients underwent dose-planning CT in prone position. After an on line CT virtual simulation, CT datasets were transferred to a dedicated treatment planning system through a DICOM network and an individualized clinical target volume (CTV) was done, including the gross tumor volume with margins (2–3 cm depending upon tumor position, defined by MRI imaging), the mesorectum and regional lymph nodes depending upon tumor location. We contoured the small bowel, the femoral heads and the bladder as critical organs on all CT slices of every patient, and we evaluated the relative dose–volume histogram on the treatment planning console. Three-dimensional plans for 3D radiotherapy were generated for a dual-energy (6 and 20MV x-rays) linear accelerator (Clinac 2100C/D) equipped with multileaf collimators (MLC). Patients were scheduled using a 3 field arrangement to include the PTV within the 95% isodose and a dose of 25 Gy in 5 fractions over 1 week was prescribed to the ICRU 62 intersection point. Adverse effects of RT were scored according to the Radiation Therapy Oncology Group (RTOG) toxicity criteria [13]. Adverse effects were defined as severe, corresponding to RTOG grade 3–4, when required hospitalization during the interval between the beginning of RT and surgery, or within 90 days.

### Surgery

Surgical planning was made on the results of restaging. Downsizing and downstaging were considered as an opportunity to perform sphincter saving surgery in those patients without sphincter involvement before treatment. Also local excision was considered a possible option in patients with a significant clinical response assessed by MRI and proctoscopy. The planned operation was discussed with the patients and a specific informed consent was obtained. A rectal resection with total mesorectal excision and bilateral nerve sparing where possible was the standard operation. In distal cancers an ultralow anterior resection with coloanal manual anastomosis or, in case of sphincters involvement, an abdomino-perineal resection were performed. All patients receiving an anastomosis underwent construction of a protecting ileostomy. Complications were classified according to the Clavien Dindo scoring [14].

### Pathology

Postsurgical pathology examination provided a macroscopic description of the mesorectum and of the former tumor-bearing area; at least four paraffin blocks were processed, and an additional larger area block was embedded. If no tumor was visible, the entire suspicious area was sliced and embedded. CRM was judged to be involved if the microscopic tumor was < 1mm from the radial resection margin. The TRG was assessed by the pathologist and scored according to a five-point system [15]. Briefly, TRG 1 was a complete tumor regression (regardless of the presence of acellular mucine lakes), and TRG 2 was a nearly complete tumor regression with extensive fibrosis; TRG 3 presented a clear evidence of residual cancer cells but with predominant fibrosis; TRG 4 was a residual of cancer cells outgrowing fibrosis; TRG 5 was the absence of regressive changes. A major response consisted of TRG 1–2 while a TRG 3–4 was classified as minor.

### Statistics

Baseline comparisons each group were performed by using chi-square test for categorical variables and Kruskal-Wallis non parametric ANOVA for age. The effect of treatment on outcomes was assessed by exact Cochrane-Armitage trend test. Multivariate analysis of the effect of the interval to surgery was performed with a logistic regression model using TRG as response variable and baseline stage and fitness as potential confounding covariates. TRG was dichotomized as TRG 1–2 (major pathological response) versus TRG 3–5. Analyses were performed with StatXact and STATA software.

## Results

Among the sixty seven patients treated with SCR, 30 (44,8%) with a clinical history of heart deficiency (relative contraindication to the fluoropirymidines), with liver or kidney deficiency were excluded from long course chemoradiation. The radiation treatment was completed in all patients. Adverse effects were registered only in 11 of 67 patients. (16,9%–10 proctitis grade 2–3, 1 enteritis grade 2 in the 2 weeks following SCR). Groups did not differ by age, gender and distance from a.v. All but one patient, with a critical morbidity who refused the operation, were referred for radical surgery. Surgery consisted of a sphincter saving procedure in 53 (80,3%) patients and a stoma operation (APR, Hartmann) in the remaining 13 (19.7%) cases. A R0 resection was achieved in 65 (98.5%) cases. Among sphincter saving procedures a low anterior resection was the most (48 patients) performed operation along with a transanal resection in 5 patients. Eighteen (27.7%) patients experienced postoperative complications with a 7.6% of Clavien-Dindo grade III events. Postoperative death occurred in 4 (6.1%) patients. All deaths occurred in critically ill patients, unfit for chemoradiation and with a high ASA score. Complication rate was higher in the group operated on between weeks 6 and 8 (Table 2).

**Table 2:** Compliance of radiotherapy and surgical procedures according to different intervals.

	< 6 wk	6–8 wk	8 wk	*p*
	n = 12 (%)	n = 19 (%)	n = 35 (%)	
**RT Toxicity**	3 (25%)	2 (10.5%)	6 (17.1%)	
**Surgical procedure (n = 66)**				0.59*
*Sphincter saving*	9 (75%)	14 (73.7%)	30 (85.7%)	
LAR	9 (75%)	14 (73.7%)	25 (71.4%)	
Transanal local resection	/	/	5 (14.3%)	
*Stoma operation*	3 (25%)	5 (26.3%)	5 (14.3%)	
Miles	2 (16.7%)	2 (10.5%)	4 (11.4%)	
Hartmann	1 (8.3%)	3 (33.3%)	1 (2.8%)	
**R0 resection**	12 (100%)	19 (100%)	34 (97.1%)	> 0.99*
**Postoperative complications**	4 (33.3%)	7 (36.8%)	7 (20%)	0.10*
Clavien Dindo				
grade I	1	4	4	
grade II	1	1	1	
grade III	1 (8.3%)	2 (22.2%)	2 (5.7%)	
**Distance from a.v.**				0.37*
≤ 5 cm	7 (58.3)	9 (47.4)	13 (37.2)	
5–8 cm	5 (41.7)	5 (26.3)	11 (31.4)	
≥ 8 cm	0 (0)	5 (26.3)	11 (31.4)	
**Deaths**	1 (8.3%)	1 (5.3%)	2 (5.7%)	

*exact chi-square test

Overall tumor regression grading indicated a poor response (grade 3–4) in 37 of 66 (56.1%) patients and a good response (grade 1–2) in the remaining 28 of 66 (42.4%)–(Table 3). Pathologic tumor downstaging occurred in 53 of 66 (80.3%). Nodal downstaging was detected in 7 of 16 (43.7%) patients deemed N+ve at pre-treatment staging and who underwent to proctectomy. Both tumor regression grading and downstaging were re-analysed dividing patients into subgroups considering the time to surgery. Rate of major patological response (TRG 1–2) significantly increased with interval to surgery ranging from 16.7% when the interval was short (< 6 weeks) to 54.3% when the interval was longer than 8 weeks. No significant difference was observed for stage, pT and pN, although the latter finding is strongly affected by the small size (Table 3).

**Table 3:** Observed outcomes by interval to surgery.

	< 6 wk	6–8 wk	8 wk	*p*
	n = 12 (%)	n = 19 (%)	n = 35 (%)	
**TRG**				0.023^
1–2	2 (16.7)	7 (36.8)	19 (54.3)	
3–5	10 (83.3)	12 (63.2)	16 (45.7)	
**pT**				0.67^
down stage	9 (75.0)	15 (78.9)	29 (82.9)	
same or upstage	3 (25.0)	4 (21.1)	6 (17.1)	
**pN**(**)			(***)	0.31*
down stage	(§)	1/5 (20.0)	6/11 (54.5)	
same or upstage	(§)	4/5 (80.0)	5/11 (45.5)	
**Stage**				0.87^
down stage	8 (66.7)	10 (52.6)	23 (62.9)	
same or upstage	4 (33.3)	9 (47.4)	12 (37.1)	

^Exact Cochrane-Armitage trend test

(§) All patients were N_0_ at baseline

*by Fisher's exact test, group 6–8 wk *vs* group > 8 wk

**only patients with baseline N>0

*** two N_1_ patients received local excision

Multivariate analysis showed that, in comparison with an interval to surgery > 8 weeks, indeed, odds ratio (OR) of response (TRG 1–2) was reduced both when interval was < 6 weeks (OR 0.11, 95% CI 0.20 to 0. 4) and when surgery occurred 6–8 weeks after SCR (OR 0.54, 95% CI 0.15 to 1.95). The OR of response was also reduced by stage 3 and unfitness, but neither one covariate reached statistical significance (Table 4).

**Table 4:** Multivariate analysis of the effect of the interval to surgery on Tumor Regression Grade (TRG).

Variable	Odds ratio (95% C.I.)	*p*
**Interval to surgery**		0.022
< 6 wk *vs* > 8 wk	0.11 (0.20 to 0.64)	
6–8 wk *vs* >8 wk	0.54 (0.15 to 1.95)	
**Stage**		0.095
3 *vs* 1–2	0.35 (0.10 to 1.20)	
**Fitness**		0.319
Unfit *vs* Fit	0.56 (0.17 to 1.77)	

C.I.: confidence interval

The reasons behind patients’ unfitness for long course chemoradiation were significantly different among the three groups mainly because of a larger rate of unfit patients in the 6 to 8 wk group. In the multivariate analysis unfit patients had a poorer outcome (OR = 0.56) that was not statistically significant (p = 0.319).

After a median follow up of 24 months, 52 patients are alive: local recurrence occurred in 4 (6.4%) and distant metastases in 3 (4.8%) patients. Six patients died: 3 because of the disease and 3 for non diseases related causes. Local recurrence occurred in 1 ypT1N0 patient (group B) treated with local excision, 3 ypN+ve patients (2 group B, 1 group C). One of these last patients had received the R1 resection. Distant metastases occurred in 1 ypT2N0 patient of group B and in 2 ypN+ve patients also from (group B). In all patients who had local or systemic relapse the TRG score was poor (grade 3–4, 1 patient TRG 5).

## Discussion

Delaying surgery after SCR induces a significant rate of tumor regression. Complete local tumor response after preoperative treatment in rectal cancer is considered an important prognostic indicator. In recent years, many efforts have been made aiming to increase response rate using hypofractionated radiotherapy or long course radiotherapy and concomitant chemotherapy with more effective drugs and regimens [16–18]. Delayed surgery (four to eight weeks interval) is usually performed after long-course preoperative chemoradiation in locally advanced rectal cancer. Delayed surgery allows a downsizing of the tumor and in many cases also a notable downstaging. This occurs with both radiotherapy doses greater than 40 Gy at a conventional fractionation and a delay of at least four weeks when there is enough time for tumor cells to die, thus obtaining a significant mass reduction. In fact Lyon R90-01 trial showed a significant increase in tumor response considering an interval of six weeks [19].

In patients undergoing classical SCR the overall treatment time (OTT) is never greater than two weeks, being the patients operated on in the week following the completion of radiotherapy. Thus the interval between SCR and surgery is too short for a significant tumor regression. Data from the Dutch trial show a small reduction in size of rectal tumors, possibly due to apoptotic death of intratumoral lymphatic cells; also a reduction of detected lymphnodes is also observed but no impact on tumor stage and number of metastatic lymphnodes is obtained [9]. Thus it is possible to assume that preoperative SCR does not have an impact on rectal cancer regression and no downstaging occurs if immediate surgery is performed. In our series, the median time from completion of SCR to surgery was 61 days (almost 8 weeks), a longer interval compared with the usual northern european approach. If compared with pre-treatment staging, ypTNM showed a downstaging in 53 of 66 (80.3%) patients.

The Stockholm III trial has randomized rectal cancer patients to either long-course radiotherapy (50 Gy), SCR with immediate surgery or SCR with delayed surgery (6–8 weeks “waiting period”). An interim analysis including 300 patients showed that delaying surgery after SCR is a feasible approach [20]. Other observational retrospective studies [10, 11, 21, 22] proved that a significant downstaging with a fair rate of pathologic complete response can be obtained in selected patients increasing the interval between completion of SCR and surgery.

All these data support the hypothesis that SCR followed by delayed surgery might produce down-staging or at least down-sizing of rectal cancer with low toxicity. Our results confirm that SCR is a well tolerated treatment. Protocol norms were strictly adhered to in all treated patients with a 16.4% of RT related complications. Delayed surgery did not modify the strategy treatment. An operation could be performed in almost all patients (one patient refused the operation because of a high morbidity risk). Despite postoperative deaths occurred in 6.1% of patients it has to be underscored that all the deaths occurred in patients with pretreatment critical clinical conditions. A major morbidity was reported in five patients (7.6%).

An effective method of “scoring” tumor response is the evaluation of tumor regression grade. It is reproducible and several scales have been described to better score the relationship between residual tumor and fibrosis induced by radiation treatment. In our experience TRG is suitable to verify the effectiveness of neoadjuvant therapy in rectal tumors [23]. Moreover TRG can help to define possible adjuvant strategies in patients treated into a neoadjuvant setting.

The delayed time to surgery in our series produces interesting results in terms of tumor regression. Tumor regression grade evaluation showed a significant local response for a treatment that is not normally, i.e. without delayed surgery, expected to induce a regression with a global response rate of 42.4% for TRG 1–2 and a 56.1% of TRG 3–4. More importantly TRG 1–2 progressively increased with the raise extension of the time interval to surgery from less than 6 weeks to more than 8 weeks, whereas the rate of poor response (TRG 3–4) was inversely reduced. Tumor regression rate is poor in patients operated any earlier than 8 weeks, but it raises/it increases if the interval is taken to more than 8 weeks. The prognostic role of the time interval to interval of surgery was confirmed at multivariate analysis, after adjustment by baseline stage and fitness (p = 0.022).

Another major issue about SCR is that it is well tolerated, a low toxicity and a good impact on local control in low risk rectal cancer patients. In our series the local recurrence rate is 6.4%, and it occurred in patients with tumours at high risk of recurrence that could be better treated with chemoradiation (N+ve, poor local response). We believe that the optimal surgery offered in all resected cases contributed to this result. In addition a disease free survival rate close to 77.5% in patients with a median 24 months follow up is an extremely encouraging result and could confirm that an effective local control (RT + surgery) might concur to provide a long term survival: a longer follow up is however needed to back up this issue.

Our study is a retrospective analysis of a single institution in which a prospective trial of delaying surgery after short RT was not planned, but introduced into a therapeutic alghoritm approved by an institutional board. The progressive increase of the delay from 2 to more than eight weeks was allowed over the time because of external scientific evidence [19]. With new evidences [5] that an increased pathologic complete response rate and downstaging occurred with an RT-surgery interval increased up to 58 days, we progressively raised the interval to more than 8 weeks. Since 2009 an interval of more than 8 weeks was costantly adopted. Notwithstanding there was just a minimum overlapping of the study periods among interval groups. Therefore there is an almost complete confounding that can not be accounted for by a multivariate analysis. This is of course another limitation of the study.

Despite this matter, this paper offers two crucial pieces of information on the issues related to the multidisciplinary approach to rectal cancer. Firstly, is that, as already shown in a different subset of patients with more advanced disease and using chemoradiation [24], pathologic downstaging is higher when surgery is performed after more than 8 weeks after the end of neoadjuvant therapy. An interval between 8 and 10 weeks may be ideal to from the increased time for the tumor to shrink and eventually disappear (complete response) and it is not long enough to increase the risk of tumor progression. Larger studies in randomized clinical trial should define the optimal interval between SCR and surgery as proposed by Evans et al [25].

The second issue is related to the feasibility of a preoperative approach to locally advanced rectal tumor by the SCR alone followed by delayed surgery. A satisfying rate of complete pathological response can be achieved in selected patients, with a treatment that carries a low toxicity and is well tolerated by the patients. Moreover, the impact on the routine activity of a radiation therapy department is lower using SCR in place of long course schedules, in order to treat a larger number of patients and to reduce patients’ waiting lists. In addition, our data support the opportunity to set SCR into a chemoradiation approach for locally advanced rectal cancer: induction or “waiting period” chemotherapy could increase the rate of tumor regression.
